# Comparative evaluation of the UMIC Colistine kit to assess MIC of colistin of gram-negative rods

**DOI:** 10.1186/s12866-019-1424-8

**Published:** 2019-03-18

**Authors:** Lucie Bardet, Liliane Okdah, Stéphanie Le Page, Sophie Alexandra Baron, Jean-Marc Rolain

**Affiliations:** 1 Aix Marseille Univ, IRD, APHM, MEPHI, IHU-Méditerranée Infection, Marseille, France; 2 IHU-Méditerranée Infection, Marseille, France

**Keywords:** Colistin, Susceptibility testing, Polymyxin, UMIC, *Enterobacteriaceae*, *Mcr-1*, Broth microdilution, Detection method, Resistance, MIC

## Abstract

**Background:**

The recent description of the first plasmid-mediated colistin-resistant gene *mcr-1*, conferring transferable and low-level resistance to colistin, raised concern about the need to implement a rapid and reliable screening method to detect colistin-resistant clinical isolates. The only valid method to assess the MIC of colistin is the broth microdilution according to the joint CLSI-EUCAST Polymyxin Breakpoints Working Group. UMIC Colistine is a ready-to-use broth microdilution kit developed to easily assess colistin MIC by proposing unitary polystyrene strips containing 11 concentrations of dehydrated colistin. Here, we evaluated the UMIC Colistine kit on 235 Gram-negative rods (176 *Enterobacterales*, including 70 harboring a *mcr* gene, and 59 non-fermentative), through comparison to the reference broth microdilution method prepared in accordance with EN ISO 20776-1:2006 standard. Reproducibility of the UMIC Colistine was assayed with the three recommended quality control strains *E. coli* ATCC 25922, *E. coli* NCTC 13846 (*mcr-1* positive), and *P. aeruginosa* ATCC 27853, as for stability testing.

**Results:**

Categorical agreement was 100% with 63.4% (*n* = 149) of colistin-resistant strains, and 36.6% (*n* = 86) of colistin-susceptible strains with both methods (S ≤ 2 μg/mL and R > 2 μg/mL). No major error or very major error was reported. Essential agreement was 94.0% (*n* = 221), and 100% for detection of colistin-resistant strains as compared to the reference method. Pearson’s correlation between UMIC Colistine and the reference method was 0.98. Reproducibility of the UMIC Colistine system was 97.8% with MICs of the quality control strains within the target ranges. However, some isolates had lower MIC with UMIC Colistine, but that did not change their categorization as colistin-susceptible, and this phenomenon should be further explored.

**Conclusions:**

The UMIC Colistine kit is an easy to perform unitary device that showed excellent results when compared to the reference method. The UMIC Colistine system is a rapid and reliable broth microdilution method that is suitable to assess the colistin MIC of clinical isolates in clinical microbiology laboratories.

## Background

The emergence of multi-drug resistant Gram-negative bacteria is a worldwide phenomenon and has led to the revival of old antibiotics as last resort treatments, including polymyxins [1]. Until 2015, all the described polymyxin-resistant genes were chromosomally encoded, including those coding for the two-component systems PmrA/PmrB, PhoP/PhoQ, and their negative regulator MgrB in the *Klebsiella pneumoniae* species [2]*.* In November 2015, the first plasmid-mediated colistin resistance gene was described and named *mcr-1* [3]. The transferable *mcr-1* gene has been detected in samples from all over the world and from various human and animal origins [4]. This discovery was followed by the description of other *mcr* genes: *mcr-2* to *mcr-8* [5–12]. This mobile colistin resistance that confers low levels of resistance with Minimal Inhibitory Concentrations (MIC) of colistin around 4 μg/mL [3] raised concerned about the capacity to detect colistin resistance in clinical microbiology laboratories [13].

Indeed, the clinical breakpoint of colistin, established by both the European Committee of Antibiotic Susceptibility Testing (EUCAST) and the Clinical and Laboratory Standards Institute (CLSI), is 2 μg/mL (resistant > 2 μg/mL and susceptible ≤2 μg/mL) for *Enterobacteriaceae*, *P. aeruginosa* and *Acinetobacter* spp. [14, 15]. More specifically, the CLSI recommends an Epidemiological Cut-off Value (ECV) and not a clinical breakpoint for the following *Enterobacteriaceae* species: *Enterobacter aerogenes, Enterobacter cloacae*, *Escherichia coli*, *Klebsiella pneumoniae* and *Raoultella ornithinolytica.*

Currently, the only available Antibiotic Susceptibility Testing (AST) method for colistin according to the joint CLSI-EUCAST Polymyxin Breakpoints Working Group is the EN ISO 20776-1:2006 standard Broth Microdilution method (BMD) [16] that has to be used with cation-adjusted Mueller-Hinton broth medium (MH2), untreated polystyrene trays without additives and colistine sulphate salt [17]. As BMD is a time-consuming technique, it is not suitable for clinical microbiology laboratories in public hospitals, and the establishment of a rapid and reliable method for colistin MIC determination is obviously warranted to control the spread of colistin resistance [13].

The UMIC Colistine kit (Biocentric, Bandol, France) was developed to easily determine colistin MIC with a ready-to-use device based on BMD method. The UMIC Colistine kit consists of unitary 12-wells polystyrene strips with 11 wells containing a range of dehydrated colistin concentrations from 0.06 to 64 μg/mL (with 2-fold dilutions between 2 consecutive wells), and one well for growth control.

Here, we evaluated the UMIC Colistine strips by comparison to the reference method prepared in accordance with the EN ISO 20776-1:2006 standard.

## Methods

### Bacterial strains

A total of 235 bacterial strains were used in this study, including 162 *Enterobacteriaceae* (77 *Escherichia coli*, 50 *Klebsiella pneumoniae*, 4 *Klebsiella oxytoca*, 18 *Enterobacter cloacae*, 4 *Enterobacter aerogenes*, 4 *Enterobacter asburiae* and 5 *Salmonella enterica*), 14 intrinsic colistin-resistant genera of *Enterobacterales* (9 *Hafnia alvei*, 1 *Proteus mirabilis*, 1 *Morganella morganii*, 1 *Providencia alcalifaciens*, 1 *Providencia rettgeri* and 1 *Serratia marcescens*), and 59 non-fermentative isolates (31 *Pseudomonas* sp., 18 *Acinetobacter* sp*.* and 10 *Stenotrophomonas maltophilia*) (Table 1) [18–23]. These microorganisms were isolated as part of standard care of patients or animals. 85 colistin-resistant isolates were well-characterized from previous studies, including 70 harboring a *mcr* gene (61 *mcr-1*, 1 *mcr-2* and 8 *mcr-3*), with MICs ranging from 4 to 64 μg/mL, and their genotype are detailed in Table 1.

**Table 1:** Colistin MICs obtained by broth microdilution comparing UMIC Colistine to the reference method (μg/mL). Discrepancies are indicated in bold

Bacterial species	Isolates	Samples origins	Genotype	Colistin MIC (μg/mL)	
				Reference	UMIC
*Escherichia coli*	NCTC 13883	Human, UK	*mcr-1*	4	4
	SE65	Human, Algeria	*mcr-1*	4	4
	117R	Human, Saudi arabia	*mcr-1*	4	4
	1R2013	Human, Saudi arabia	*mcr-1*	8	8
	1R 2104	Human, Saudi arabia	*mcr-1*	8	4
	44A	Human, Saudi arabia	*mcr-1*	4	4
	6R	Human, Saudi arabia	*mcr-1*	4	4
	85R	Human, Saudi arabia	*mcr-1*	4	8
	95R	Human, Saudi arabia	*mcr-1*	4	8
	96R	Human, Saudi arabia	*mcr-1*	8	8
	134R	Human, Saudi arabia	*mcr-1*	4	4
	143R	Human, Saudi arabia	*mcr-1*	8	8
	LH121	Human, Laos	*mcr-1*	4	4
	LH140	Human, Laos	*mcr-1, phoQ* E375K	8	8
	LH257	Human, Laos	*mcr-1*	16	8
	LH57	Human, Laos	*mcr-1, phoQ* E375K	4	4
	LH1	Human, Laos	*mcr-1*	4	4
	LH30	Human, Laos	*mcr-1*	4	4
	LH345.2	Human, Laos	*mcr-1*	4	4
	TH214	Human, Thailand	*mcr-1*	4	8
	TH99	Human, Thailand	*mcr-1*	16	16
	TH169.1	Human, Thailand	*mcr-1*	4	4
	TH259.1	Human, Thailand	*mcr-1*	4	4
	TH33.1	Human, Thailand	*mcr-1*	4	4
	TH44.1	Human, Thailand	*mcr-1*	4	4
	TH66.1	Human, Thailand	*mcr-1*	4	8
	TH134.1	Human, Thailand	*mcr-1*	4	4
	FHM128.1	Human, France	*mcr-1*	4	4
	FHM66.1	Human, France	*mcr-1*	4	4
	P4.5 t3 (4)	Pig, Lebanon	*mcr-1*	8	4
	P1.2 (16)	Pig, Lebanon	*mcr-1*	4	4
	P1.38 (18)	Pig, Lebanon	*mcr-1*	4	4
	P1.5 t2 (8)	Pig, Lebanon	*mcr-1*	4	4
	P2.12 (13)	Pig, Lebanon	*mcr-1*	4	4
	P2.13 t1 (11)	Pig, Lebanon	*mcr-1*	4	4
	P2.13 t2 (12)	Pig, Lebanon	*mcr-1*	4	4
	P2.3 t2 (15)	Pig, Lebanon	*mcr-1*	4	4
	P2.6 (14)	Pig, Lebanon	*mcr-1*	4	4
	P4.21 t1 (7)	Pig, Lebanon	*mcr-1*	4	4
	P4.5 t1 (1)	Pig, Lebanon	*mcr-1*	4	4
	12	Environmental, Algeria	*mcr-1*	8	8
	14	Environmental, Algeria	*mcr-1*	8	8
	28	Environmental, Algeria	*mcr-1*	8	8
	31	Environmental, Algeria	*mcr-1*	8	8
	3	Environmental, Algeria	*mcr-1*	4	4
	5	Environmental, Algeria	*mcr-1*	4	4
	10	Environmental, Algeria	*mcr-1*	4	8
	15	Environmental, Algeria	*mcr-1*	4	4
	39	Environmental, Algeria	*mcr-1*	4	4
	MCR-2	Pig, Belgium	*mcr-2*	4	4
	16	Environmental, Algeria	*mcr-3*	8	4
	8	Environmental, Algeria	*mcr-3*	4	8
	FHA102	Human, France	*pmrB* A159V	16	16
	FHM19	Human, France	*pmrB* P7-Q12 del (6 aa)	16	8
	FHA113	Human, France	*pmrB* T156K	8	8
	NH94	Human, Nigeria	*pmrB* I92 insertion	16	16
	LH345.1	Human, Laos		4	4
	LH53	Human, Laos		4	4
	TH176	Human, Thailand		8	8
	TH169.5	Human, Thailand		4	4
	LB4	Human, France		8	8
	235	Chicken, Algeria	*mcr-1*	4	4
	P6	Pig, Laos	*mcr-1*	4	4
	P10	Pig, Laos	*mcr-1*	4	8
	P17	Pig, Laos	*mcr-1*	8	4
	P7	Pig, Laos		4	4
	ATCC 25922	Human, Unknown		0.5	0.25
	ATCC 35218	Unknown		1	0.5
	EC1	Human, France		0.5	0.25
	EC2	Human, France		0.5	0.5
	EC3	Human, France		0.5	0.25
	EC4	Human, France		1	0.5
	LH165S*	Human, Laos		**1**	**0.25**
	TH77S	Human, Thailand		0.5	0.25
	282S	Chicken, Algeria		1	1
	161	Chicken, Algeria		1	0.5
	NDM-1	Human, Israël		0.5	0.25
*Klebsiella pneumoniae*	FHA60	Human, France	*mcr-1*	16	16
	FHM128	Human, France	*mcr-1*	16	8
	119R	Human, Saudi arabia	*mcr-1*	8	8
	LH131	Human, Laos	*mcr-1, mgrb* stop	32	32
	LH61	Human, Laos	*mcr-1, mgrB* sub A14S	32	32
	LH17	Human, Laos	*mcr-1, pmrB* T157P	32	32
	LH92	Human, Laos	*mcr-1*	16	16
	LH94	Human, Laos	*mcr-3*	32	32
	TH68	Human, Thailand	*mcr-3*	64	64
	LH102	Human, Lao	*mcr-3*	16	32
	LH375	Human, Lao	*mcr-3*	16	16
	TH114	Human, Thailand	*mcr-3*	16	16
	TH164	Human, Thailand	*mcr-3*	16	16
	LB1	Human, France	*mgrB* Stop	64	64
	FHM169	Human, France	*mgrB* Stop	16	16
	LH12	Human, Laos	*mgrB* Stop	32	32
	TH28	Human, Thailand	*mgrB* IS2	32	32
	TH54	Human, Thailand	*pmrB* T157P	16	16
	TH224	Human, Thailand	*pmrB* T157P	8	16
	TH205	Human, Thailand		8	8
	FHM120	Human, France		32	32
	FHA105	Human, France		64	64
	FHM77	Human, France		16	16
	LB3	Human, France		> 64	> 64
	SB11R	Human, France		> 64	> 64
	SB12R	Human, France		> 64	> 64
	LH140	Human, Laos		64	64
	KP1PC	Human, France		16	16
	KP2PC	Human, France		16	16
	4321	Human, UK		32	32
	K39	Human, Greece		> 64	64
	K76	Human, Greece		> 64	> 64
	1172/0	Human, Greece		32	32
	7E	Human, Greece		32	32
	18E	Human, Greece		16	16
	28E	Human, Greece		16	16
	9980	Human, Greece		16	16
	K77	Human, Greece		16	16
	1E	Human, Greece		8	8
	KAT3	Human, Greece		8	8
	2017–10	Human, Greece		1	0.5
	1678	Human, Greece		0.5	1
	56	Human, Greece		0.5	0.25
	K72	Human, Greece		0.5	0.5
	KP1	Human, France		0.5	0.25
	KP6	Human, France		0.5	0.25
	TH28S	Human, Thailand		0.5	0.25
	CIP 82.91	Unknown		0.25	0.25
	LB2*	Human, France		**1**	**0.25**
	ATCC 700603	Human, UK		0.5	0.25
*Klebsiella oxytoca*	FHA41	Human, France	*mgrB* IS1	32	64
	FHA124	Human, France		16	32
	TH44*	Human, Thailand		**0.5**	**0.125**
	KOX1	Human, France		0.5	0.25
*Enterobacter aerogenes*	EA1509E	Human, France	*pmrA* G157A	> 64	> 64
	SB7R	Human, France		32	16
	EAE1	Human, France		0.5	0.25
	EAE2	Human, France		1	0.5
*Enterobacter asburiae*	LH74	Human, Laos		> 64	> 64
	TH66	Human, Thailand		32	64
	1502	Human, France		0.5	0.25
	1503	Human, France		0.5	0.25
*Enterobacter cloacae*	SB1	Human, France	*mcr-1*	4	4
	NH131	Human, Nigeria		> 64	> 64
	NH132	Human, Nigeria		> 64	> 64
	NH52	Human, Nigeria		> 64	> 64
	SB5R	Human, France		> 64	> 64
	SB6R	Human, France		> 64	> 64
	SB10R	Human, France		> 64	64
	SB4R	Human, France		64	64
	TH66	Human, Thailand		32	32
	SB3R*	Human, France		**2**	**0.25**
	SB2R	Human, France		2	2
	SB5S	Human, France		2	1
	SB1S*	Human, France		**1**	**0.25**
	SB2S*	Human, France		**1**	**0.25**
	SB3S*	Human, France		**1**	**0.25**
	P7698*	Human, France		**1**	**0.25**
	NH151	Human, Nigeria		0.5	0.25
	NH74	Human, Nigeria		0.5	0.25
*Salmonella enterica*	100RC3	Human, Saudi Arabia	*pmrB* deletion (12aa)	8	8
	65R	Human, Saudi Arabia	*pmrB* deletion (12aa)	8	8
	122R	Human, Saudi Arabia		1	0.5
	108R	Human, Saudi Arabia		1	0.5
	10A	Human, Saudi Arabia		1	1
*Hafnia alvei*	B42	Bird, France		8	8
	P516	Human, France		8	8
	A63	Bird, France		4	4
	B11	Bird, France		4	8
	B21	Bird, France		4	8
	B47	Bird, France		4	4
	B59	Bird, France		4	4
	B02	Bird, France		4	4
	B04	Bird, France		4	4
*Morganella morgannii*	FHA60	Human, France		> 64	> 64
*Proteus mirabilis*	NDM-1	Human, Israel		> 64	> 64
*Providencia alcalifaciens*	TH66	Human, Thailand		> 64	> 64
*Providencia rettgeri*	TH66	Human, Thailand		> 64	> 64
*Serratia marcescens*	P6	Chicken, Algeria		> 64	> 64
*Stenotrophomonas maltophilia*	SM10	Human, France		> 64	> 64
	SM7	Human, France		32	16
	SM8	Human, France		32	32
	SM9	Human, France		32	32
	SM6	Human, France		16	16
	SM4	Human, France		8	8
	SM5	Human, France		8	8
	SM2	Human, France		4	4
	SM3	Human, France		4	4
	SM1	Human, France		1	1
*Pseudomonas aeruginosa*	FHM-PACOLR1	Human, France		> 64	> 64
	ATCC 27853	Human, unknown		1	1
	FHM_PA7	Human, France		1	1
	FHM-PA2	Human, France		2	2
	FHM-PA3	Human, France		2	2
	FHM-PA4	Human, France		1	1
	FHM-PA5	Human, France		1	1
	FHM-PA6	Human, France		1	1
	PA1	Human, France		0.5	0.5
	PA2	Human, France		1	1
	PA3	Human, France		1	2
	PA4	Human, France		0.5	0.25
	PA5	Human, France		1	1
	PA6	Human, France		0.5	0.25
	PA7	Human, France		1	1
*Pseudomonas putida*	AEM06	Environmental, France		1	0.5
	AEM10	Environmental, France		1	0.5
	AEM15	Environmental, France		1	0.5
	ETP11	Environmental, France		0.5	0.5
	AEM08 B	Environmental, France		0.5	0.5
	AEM12	Environmental, France		0.5	0.5
	AEM13	Environmental, France		0.5	0.5
	AEM16	Environmental, France		0.5	0.5
	AEM17 A	Environmental, France		0.5	0.5
	AEM17 B	Environmental, France		0.5	0.25
	AEM19	Environmental, France		0.5	0.25
	PLC009	Environmental, France		0.5	0.25
*Pseudomonas stutzeri*	AEM05	Environmental, France		0.25	0.5
*Pseudomonas sp.*	AEM08 A	Environmental, France		1	0.5
	AEM07	Environmental, France		0.5	0.5
	AEM20	Environmental, France		0.25	0.5
*Acinetobacter baumannii*	ABIsac_ColiR	Human, France	*pmrA* E8D	64	64
	AB3	Human, France		4	4
	AB9*	Human, France		**2**	**0.25**
	4322	Human, UK		1	1
	Big	Human, Iran		1	0.5
	Small	Human, Iran		1	0.5
	AB1	Human, France		1	0.5
	AB2	Human, France		1	0.5
	AB4	Human, France		1	0.5
	AB5	Human, France		1	0.5
	NDM-1*	Human, Lebanon		**1**	**0.25**
	AB6*	Human, France		**1**	**0.25**
	AB8*	Human, France		**1**	**0.25**
	AB10*	Human, France		**1**	**0.25**
	CR17	Human, Spain		0.5	0.5
*Acinetobacter nosocomialis*	ABG13S	Human, Spain		1	0.5
*Acinetobacter pitti*	G867*	Human, France		**1**	**0.25**
*Acinetobacter* sp*.*	LH213	Human, Laos		1	1

*Those strains have been tested in triplicate, details are explained in the text

The three Quality Control (QC) strains recommended by EUCAST for colistin susceptibility testing, *Escherichia coli* ATCC 25922, *P. aeruginosa* ATCC 27853 and *E. coli* NCTC 13846 (*mcr-1* positive) were included in the study.

### Broth microdilution plates preparation

The BMD reference method was prepared accordingly to the EN ISO 20776-1:2006 standard, with a stock solution of colistin prepared from colistin sulphate salt (MP Biomedicals, Illkirch, France) that was adjusted accordingly to the CLSI 2017 M100 guidelines [15]. The BBL™ Mueller-Hinton II Broth (Becton-Dickinson, Heidelberg, Germany) was used as MH2 for reference method and prepared following the manufacturer’s instructions. The stock solution of colistin was diluted in the MH2 medium in order to fill the 96-well polystyrene plates (ref. 3799, Corning, Hazebrouck, France) following the same scheme of UMIC Colistine strips (0.06 to 64 μg/mL of colistin), with a growth control well containing only MH2 medium. Stock solution and plates were freshly prepared every test day.

### Colistin MIC testing

Each isolate was inoculated in parallel in both systems from the same 0.5 McFarland (McF) suspension in such a way as to obtain the same final inoculum of 5 × 10^5^ CFU/mL (Colony Forming Unit / mL) or 5 × 10^4^ CFU/well, by a 200-fold dilution. For UMIC Colistine, the 0.5 McF suspension was directly diluted in one of the MH2 tubes provided with the kits, of which 100 μL were added in each well of a unitary strip. For the reference method, an intermediate dilution was performed in the prepared MH2 medium then diluted in the 12 wells of a row of a freshly prepared plate. The QC strain *E. coli* NCTC 13846 was used as quality control each day of testing.

Results were read after incubation in aerobic atmosphere at 35 ± 1 °C for 18 ± 2 h, directly or after adding 50 μL of a prepared 5 mg/mL iodonitrotetrazolium chloride solution (Sigma-Aldricht, Illkirch, France), and could also be analyzed using ELX808 Ultra Microplate Readers (Biotek Instruments, Winooski, USA).

### Reproducibility of UMIC Colistine

The reproducibility of the UMIC Colistine kit was assessed by testing the three QC strains in triplicate on 5 different days by 3 different laboratories, resulting in 45 values for each strain. Subcultures of each day’s samples were performed from the same primary culture as recommended.

### Data analysis

Data were analyzed according to the EN ISO 20776-2:2007 standard [24] and EUCAST guidelines (resistant > 2 μg/mL or susceptible ≤2 μg/mL), using the MIC obtained with the prepared BMD method as reference MIC.

Categorical Agreement (CA, same clinical categorization), Essential Agreement (EA, MIC within ±1 doubling dilution from the reference MIC), Major Errors (ME, false resistant) and Very Major Errors (VME, false susceptible) were calculated by comparing the MICs obtained with UMIC Colistine to the reference MICs. Isolates with discrepant results were retested at least twice, and if not corrected, the values obtained from the first assay were kept. To be validated, the UMIC Colistine device should met the following criteria: CA ≥ 90%, EA ≥ 90%, ME ≤3% and VME ≤ 3%.

The correlation between the two systems was calculated using the Pearson method (value 128 was retained when the MIC was > 64 μg/mL).

The expected colistin MIC ranges for the QC strains are 0.25–1 μg/mL for *E. coli* ATCC 25922, 0.5–2 μg/mL for *P. aeruginosa* ATCC 27853 and 4 μg/mL with occasionally accepted values 2 and 8 μg/mL for *E. coli* NCTC 13846 [25]. The MIC values obtained for the three QC strains have to be in the acceptable ranges for ≥95% and reproducibility has to be comprised between ± one dilutions of the mode for ≥95% of the MIC results.

### Stability of the UMIC Colistine

Stability assays on the UMIC Colistine strips were performed using the three QC strains. Stress test or shipping stability was assayed on strips that were previously incubated at 40 °C during 1 and 2 days, 1, 2, 3 and 4 weeks. Stability in use of UMIC Colistine was assayed by opening the package of the strips 1, 3, 6 and 24 h before use.

UMIC Colistine strips that were stored as recommended by the manufacturer were used as control. All tests were performed in triplicate and for each assay the same inoculum was used on all the strips tested for each strain.

## Results

### MIC results

**Fig. 1:**
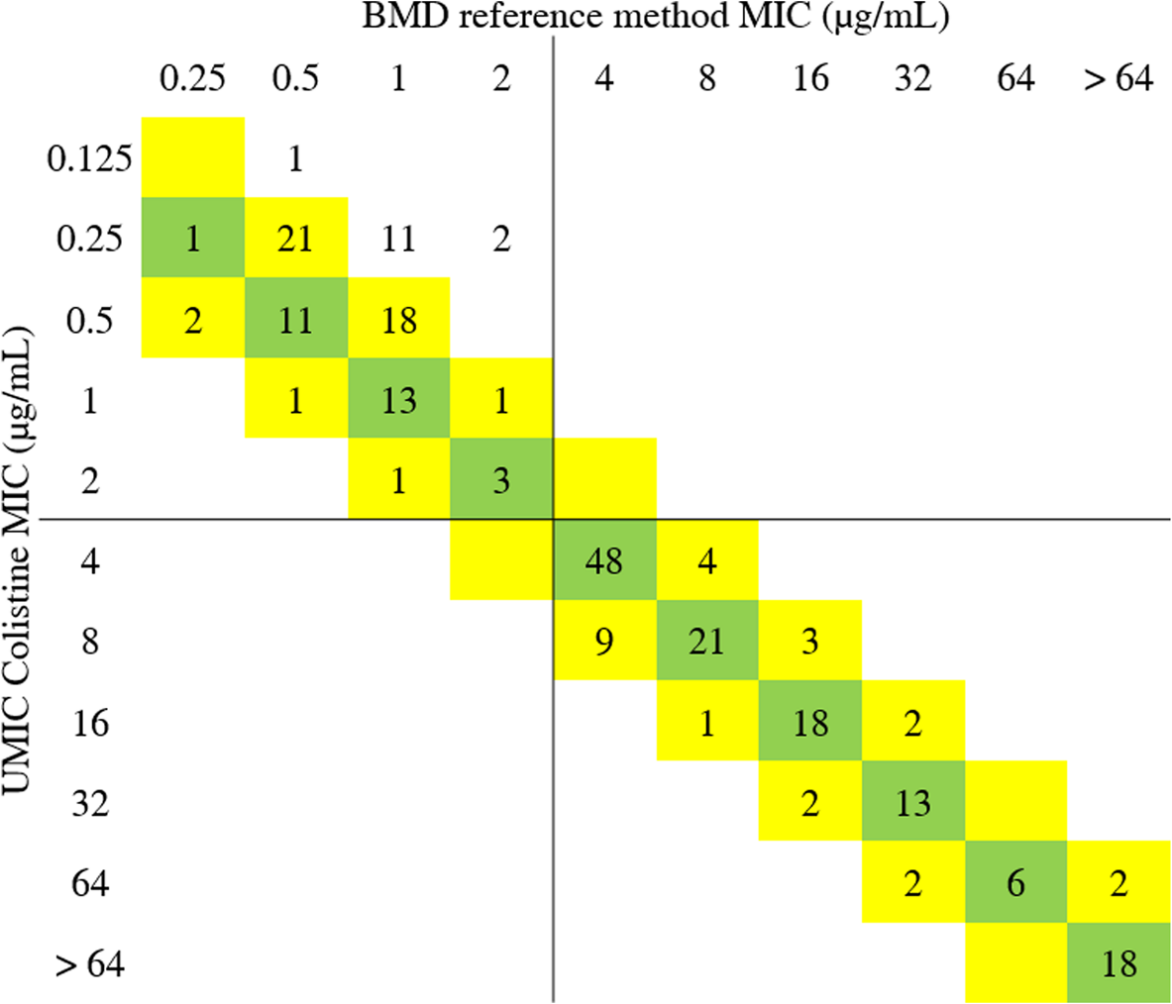
Categorical agreements for colistin MIC values between UMIC Colistine and reference method. Green shade is for identical values, and yellow shades for values within the essential agreement. Breakpoints are indicated with lines, according to CLSI recommendations (R > 2 μg/mL S ≤ 2 μg/mL)

The colistin MICs obtained for all isolates are summarized in Table 1: Categorical agreement was 100% with 63.4% (*n* = 149) of colistin-resistant strains, and 36.6% (*n* = 86) of colistin-susceptible strains with both methods, as highlighted in Fig. 1. No major error nor very major error was reported.

Essential agreement was 94% (*n* = 221), with 64.7% (*n* = 152) identical values, and was 100% for colistin-resistant strains, including all the strains harboring the *mcr* genes. Indeed, fourteen strains classified as susceptible presented a lower MIC with UMIC Colistine (Fig. 1), and were distributed into different species: 1 *E. coli*, 1 *K. pneumoniae*, 1 *K. oxytoca, 5 E. cloacae,* 5 *A. baumannii* and 1 *A. pitti* (Table 1). Those strains were tested in triplicate, resulting in the reproducibility of the discrepancies, giving MICs of 1 or 2 μg/mL with reference method and 0.25 μg/mL with UMIC Colistine, except for *K. oxytoca* TH44 which gave a MIC of 0.5 or 1 μg/mL with reference method and 0.125 μg/mL with UMIC Colistine. However, their clinical categorization did not change as all the results obtained classified those isolates as colistin-susceptible and the correlation between UMIC Colistine and the reference method was 98.0% (Pearson’s r = 0.9801).

### Reliability of UMIC Colistine

The reproducibility and quality performance of the UMIC Colistine system were both 97.8%, with only 3 values out of range for *P. aeruginosa* ATCC 27853 strain (MIC = 0.25 μg/mL). The QC strain *E. coli* NCTC 13846 always gave the recommended MIC of 4 μg/mL, except for 3 results at 2 μg/mL, which is occasionally acceptable according to EUCAST [26].

UMIC Colistine strips remained stable until 4 weeks of incubation at 40 °C and until 24 h after opening the package, obtaining the same MICs within the acceptable ranges at the different time points for the three QC strains as compared to control strips.

### Usability

Manual preparation of BMD plates was time-consuming (about 1 h per day), needed a large amount of sterile material (sterilized MH medium, plates, colistin solution, etc.) and is a source of errors as it requires many steps: weighing, dissolving, diluting, distributing. The UMIC Colistine kit provided a complete assay that only requires traditional laboratory equipment. It is rapid and easy to use, and the skipped wells are mostly avoided as it is easy to check if the wells are empty or filled with a volume of 100 μL. The results reading was clear with flat-bottom wells.

## Discussion

The need to implement a protocol to screen colistin-resistant isolates in clinical microbiology laboratories is urgent and requires a rapid and reliable method to replace BMD [27]. UMIC Colistine is easy to use and our study demonstrated its reliability to assess colistin susceptibility, as it could detect all the colistin-resistant isolates, notably all of the 70 *mcr*-positive strains tested. All the accuracy criteria were met, UMIC Colistine exhibited a high reproducibility, and quality performances where excellent even when testing strips that were stored at 40 °C to reflect the real conditions that could occur during storage and shipping of the device.

Discrepant results were obtained for some strains, mostly on *Acinetobacter* sp. and *Enterobacter* sp., but without impact on their categorization as colistin-susceptible. Those differences could be due to technical variations for some unknown reason, notably during the manual preparation of BMD with the possible loss of colistin, or to a particular phenotype of those isolates exhibited by the different features of the devices that could lead to the adhesion to the polystyrene surface of the wells. Indeed, microplates used for the reference method are tissue-culture treated, that should not impact on the colistin MIC, when UMIC Colistine strips are made of untreated polystyrene, corresponding to the recommendations on colistin susceptibility testing [17]. Additionally, the impact of the MH2 used was explored by testing the cation concentration of MH2 media used in this study. The results obtained were similar and acceptable, and the impact of the medium was eliminated: concentrations of Ca^2+^ were 22.1 mg/L for prepared MH2 and 22.06 mg/L for MH2 tubes provided with UMIC, and Mg^2+^ were 11.4 mg/L for both, when the required values are 20–25 mg/L for Ca^2+^ and 10–12.5 mg/L for Mg^2+^ according to EN ISO 20776-1:2006 standard.

Recently, the UMIC Colistine kit was evaluated together with other commercial colistine susceptibility testing devices in two studies that exhibited categorical agreements of 92 and 91.9% [26, 28]. The study performed by EUCAST obtained an essential agreement of 82% on 75 strains, with 3 VME and 3 ME, but the low number of isolates and species tested and the fact that the UMIC Colistine was not assayed with the same inoculum of the reference method can explain this lower agreement [26]. More recently, the evaluation of Jayol et al. [28] obtained 15 VME when testing 185 Gram-negative isolates: 2 *H. alvei,* 1 *K. pneumoniae*, 4 *E. coli,* 4 *S. enterica* and 4 *S. maltophilia*, including 5 *mcr*-positive isolates. There is no information on the essential agreement which seems to be over the 90% required. Concerning results were found for *S. maltophilia* isolates with high MIC but we did not find those discrepancies when testing 10 *S. maltophilia* strains. Moreover, all the *H. alvei* and *mcr-*positive isolates tested in our study were found to be colistin-resistant with UMIC Colistine. As the MH2 broth medium used for the reference method in these two studies was different from the MH2 medium used in our study, certainly explaining the differences observed in the results, it could be interesting to perform further studies evaluating different MH2 broth media for colistin susceptibility testing.

Finally, the UMIC Colistine kit has to be assayed on colistin-heteroresistant strains, that are also difficult to detect and often classified as susceptible [29].

## Conclusion

The UMIC Colistine kit consists of an easy to perform technique that gave excellent results. UMIC Colistine is a reliable method to perform broth microdilution and assess the colistin MIC of clinical isolates in clinical microbiology laboratories.

## Abbreviations

AST: Antibiotic Susceptibility Testing
ATCC: American Type Cultures Collection
BMD: Broth Microdilution
CA: Categorical Agreement
CFU: Colony Forming Unit
CLSI: Clinical and Laboratory Standards Institute
EA: Essential Agreement
ECV: Epidemiological Cut-off Values
EN: European Norm
EUCAST: European Committee of Antibiotic Susceptibility Testing
ISO: International Organization for Standardization
McF: McFarland
ME: Major Error
MH2: Mueller-Hinton
MIC: Minimal Inhibitory Concentration
NCTC: National Collection on Type Culture
VME: Very Major Error
